# Neural Basis of Increased Cognitive Control of Impulsivity During the Mid-Luteal Phase Relative to the Late Follicular Phase of the Menstrual Cycle

**DOI:** 10.3389/fnhum.2020.568399

**Published:** 2020-11-12

**Authors:** Jin-Ying Zhuang, Jia-Xi Wang, Qin Lei, Weidong Zhang, Mingxia Fan

**Affiliations:** ^1^Faculty of Education, East China Normal University, Shanghai, China; ^2^School of Psychology and Cognitive Science, East China Normal University, Shanghai, China; ^3^Department of Physics, Shanghai Key Laboratory of Magnetic Resonance, East China Normal University, Shanghai, China

**Keywords:** cognitive control, impulsivity, menstrual phase, dlPFC, dorsal striatum

## Abstract

Hormonal changes across the menstrual cycle have been shown to influence reward-related motivation and impulsive behaviors. Here, with the aim of examining the neural mechanisms underlying cognitive control of impulsivity, we compared event-related monetary delay discounting task behavior and concurrent functional magnetic resonance imaging (fMRI) revealed brain activity as well as resting state (rs)-fMRI activity, between women in the mid-luteal phase (LP) and women in the late follicular phase (FP). The behavioral data were analyzed and related to neural activation data. In the delay discounting task, women in the late FP were more responsive to short-term rewards (i.e., showed a greater discount rate) than women in the mid-LP, while also showing greater activity in the dorsal striatum (DS). Discount rate (transformed *k*) correlated with functional connectivity between the DS and dorsal lateral prefrontal cortex (dlPFC), consistent with previous findings indicating that DS-dlPFC circuitry may regulate impulsivity. Our rs-fMRI data further showed that the right dlPFC was significantly more active in the mid-LP than in late FP, and this effect was sensitive to absolute and relative estradiol levels during the mid-LP. DS-dlPFC functional connectivity magnitude correlated negatively with psychometric impulsivity scores during the late FP, consistent with our behavioral data and further indicating that relative estradiol levels may play an important role in augmenting cognitive control. These findings provide new insight into the treatment of conditions characterized by hyper-impulsivity, such as obsessive compulsive disorder, Parkinson disease, and attention deficit hyperactivity disorder. In conclusion, our results suggest that cyclical gonadal hormones affect cognitive control of impulsive behavior in a periodic manner, possibility via DS-dlPFC circuitry.

## Introduction

It has been posited that observed influences of menstrual cycle phase in women on reward-related impulsivity reflect alterations in brain dopamine function and thus downstream effects of those alterations on dopamine efferent targets in the basal ganglia and frontal cortex (Xiao and Becker, 1994; Jackson et al., 2006). These circuits are critically involved in temporal and reward processing (McClure et al., 2004; Kim et al., 2012); they are organized by gonadal steroid hormones during early development and are modulated by these hormones in adults (Xiao and Becker, 1994; Jackson et al., 2006). When estradiol levels are highest during the late follicular phase (FP), women have been shown to be more responsive to rewarding substances, such as cocaine (Terner and De Wit, 2006), and reward-related activation in the mesolimbic system has been shown to be enhanced in the late FP, compared with that in the mid-luteal phase (LP) when progesterone levels peak (Dreher et al., 2007). Self-reported liking of smoked cocaine is greater during the late FP than the mid-LP (Sofuoglu et al., 1999; Evans and Foltin, 2006). Animal studies have also provided evidence of estrogenic modulation of reward-related impulsivity. For example, female rats exhibit their maximal cocaine self-administration levels shortly after estradiol peaks, and exogenous administration of estradiol enhances the acquisition of cocaine self-administration in ovariectomized female rats (Lynch et al., 2001; Jackson et al., 2006). These findings suggest that the proclivity of female mammals to wait for a higher reward is reduced in the late FP relative to the mid-LP.

In contrast, progesterone may play a role in reducing impulsive behavior by favoring more cognitive control. Progesterone is a female gonadal hormone that is often included in hormonal contraceptives and drugs used to maintain pregnancies (Jones et al., 2005). Exogenously delivered progesterone can alleviate stimulant abuse in animals (Anker et al., 2009; Zlebnik et al., 2014) and may support stimulant use cessation in humans (Evans and Foltin, 2006, 2010; Quinones-Jenab and Jenab, 2010; Fox et al., 2013; Carroll and Lynch, 2016; Swalve et al., 2016). Furthermore, progesterone, or its metabolite allopregnanolone, reduce stress and impulsive behavior as measured by the Stroop test in humans (Milivojevic et al., 2016). Neuroimaging studies have demonstrated that increases in progesterone levels from the late FP to the mid-LP result in changes in activity in several prefrontal cortex regions (Dreher et al., 2007; Van Wingen et al., 2008; Ossewaarde et al., 2011; Marečková et al., 2012). Thus, the relatively high progesterone and low estradiol levels during the mid-LP may affect the functioning of brain regions involved in cognitive control in a manner that results in reduced impulsivity.

The main estrogen receptor is highly expressed in the amygdala (Wharton et al., 2012), which sends glutamatergic efferents to the striatum, especially the dorsal striatum (DS), a crucial brain area in the reward system related to impulsivity (Yager et al., 2015). Cummings et al.’s results (2014) suggested that estradiol enhances dopamine release in the DS, but not ventral striatum (VS), in female rats. A recent study showed that women’s responses to drug cues in the DS, specifically in the putamen, were modulated by menstrual phase (Franklin et al., 2019).

A large body of evidence indicates that the dorsolateral prefrontal cortex (dlPFC) plays an important role in cognitive control (Sheline et al., 2010; Cieslik et al., 2013). Moreover, the quality of communication between the DS and dlPFC may modulate cognitive control of impulsivity. A neural circuit linking the right caudate nucleus head and putamen with the dlPFC has been identified as a cognitive loop (Rotge et al., 2008). Commonly, patients with altered frontal-striatal function—such as in obsessive compulsive disorder (Heuvel et al., 2005), Parkinson disease (Williamsgray et al., 2007), and Huntington’s chorea (Watkins et al., 2000)—exhibit executive functioning deficits. Reduced resting state (rs)-functional connectivity between the right caudate and dlPFC has been associated with cognitive control deficits in internet gaming disorder (Yuan et al., 2016). Remarkably, in a functional magnetic resonance imaging (fMRI) study examining rs-functional connectivity in women smokers, Wetherill et al. (2016) found that, compared to data obtained during the LP, when in the FP of their menstrual cycles women had lower rs-functional connectivity between cognitive control areas (dorsal/subgenual anterior cingulate cortex and medial orbitofrontal cortex) and a reward-related region (i.e., the VS) as well as less cognitive control over their smoking behavior.

Humans have a tendency for delay discounting, a phenomenon wherein the value of a delayed rewards are over-discounted relative to that of a reward that can be obtained immediately or sooner (Frederick et al., 2002; Green and Myerson, 2004). Delay discounting has been conceptualized as an index of impulsivity (Staubitz, Lloyd, and Reed). Theoretically, the more a person discounts the value due to its delay, the more impulsive their choice is considered. Patients with conditions that affect brain dopamine systems, namely Parkinson disease and obsessive compulsive disorder, have been shown to exhibit more pronounced delay discounting than healthy and neurotypical people (Guttman and Seeman, 1985; Volkow et al., 1993; Hesse et al., 2005). This intensified preference for selecting of smaller, sooner rewards over larger, later rewards in these populations has been termed a Now bias (Smith et al., 2014).

**FIGURE 1:**
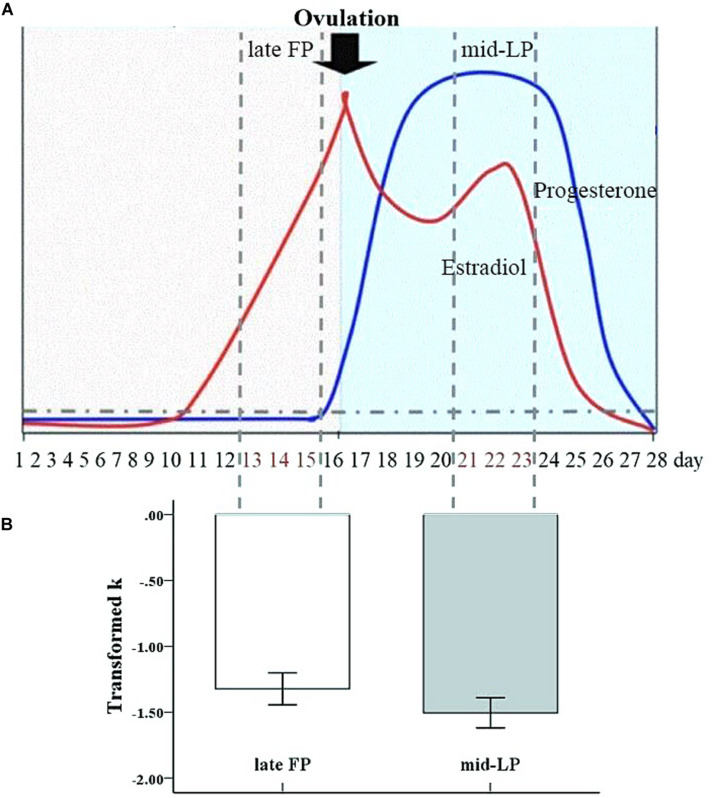
Transformed discount rate (*k*) by menstrual phase. The mean transformed *k* value was significantly greater during the late FP than during the mid-LP (error bars indicate the standard error of the mean).

The aim of the present study was to investigate the neural mechanisms underlying the modulatory effects of menstrual cycle phase on cognitive control of impulsivity with an event-related monetary delay discounting behavioral task and a rs-fMRI study. We chose to compare behavioral and imaging results between the late FP, when there are high estradiol levels with low progesterone levels, and the mid-LP, when both estradiol and progesterone levels are relatively high (Figure 1). We hypothesized that, compared to women in the mid-LP, women in the late FP would show a greater Now bias in the delay discounting task concomitant with higher activity in the DS. Furthermore, we hypothesized that a brain regions related to cognitive control, including the dlPFC, would be more active during the mid-LP, leading to the behavioral acceptance of later, larger rewards. Finally, we hypothesized that DS-dlPFC functional connectivity may play an important role in regulating impulsivity. To test our hypotheses, choices in the delay discounting task (McClure et al., 2004), performed during fMRI scanning, were compared between women in the late FP and women in the mid-LP (task fMRI study). Because fMRI signal fluctuations at rest contain information about functional network architecture (Fox et al., 2005; Fox and Raichle, 2007; Biswal, 2012), we also compared rs-functional connectivity between these two groups (rs-fMRI study) and correlated the connectivity data to hormone level data.

## Task fMRI Study

### Methods

#### Participants

A cohort of 24 healthy, right-handed, female undergraduates (mean age [± standard deviation (SD)], 22.79 ± 1.44 years; range 20–25) were enrolled. The participants were recruited from a larger, common subject pool with certain inclusion/exclusion criteria, only individuals who were heterosexual, reported having a 28- to 30-day menstrual cycle, and did not take any form of hormones in the previous 3 months were included. Because the criteria for these other studies included heterosexuality as an inclusion criteria, the participants in the current study were exclusively heterosexual. However, it is important to note that this criteria was incidental (and otherwise irrelevant) for the research questions addressed in the current study. The participants were asked to come to the laboratory on two separate occasions (late FP and mid-LP) to complete an intertemporal choice task. The tasks were the same the two testing times, but we did not state this explicitly to the participants.

We used the backward counting method to predict each participant’s next menstrual onset, late FP (14–16 days prior to the predicted menstrual onset), and mid-LP (6–8 days prior to the predicted menstrual onset). This method has been successfully used to predict other effects of theoretical interest (Durante et al., 2011; Zhuang and Wang, 2014). If a woman’s next predicted menstrual onset was 8–14 days away, she was scheduled to complete the mid-LP testing first (*N* = 10); otherwise, she was scheduled to complete the late FP testing first (*N* = 14).

All participants had normal or corrected-to-normal vision. No participants reported a history of a psychiatric disorder or current use of a psychoactive medication. The protocol was reviewed and approved by the Ethics Committee of the local University and the study was conducted in accordance with the Declaration of Helsinki. Written informed consent was obtained from all participants, and they were compensated 50 RMB per hour.

#### Behavioral Task and Procedure

Prior to scanning, the subjects were presented with a 9-rung ladder scale. They were instructed to place themselves on the ladder that ranged from 1 (lowest rung) to 9 (highest rung) based on where they stand compared to others in terms of family economic level and social status level, respectively (Adler et al., 1994).

**FIGURE 2:**
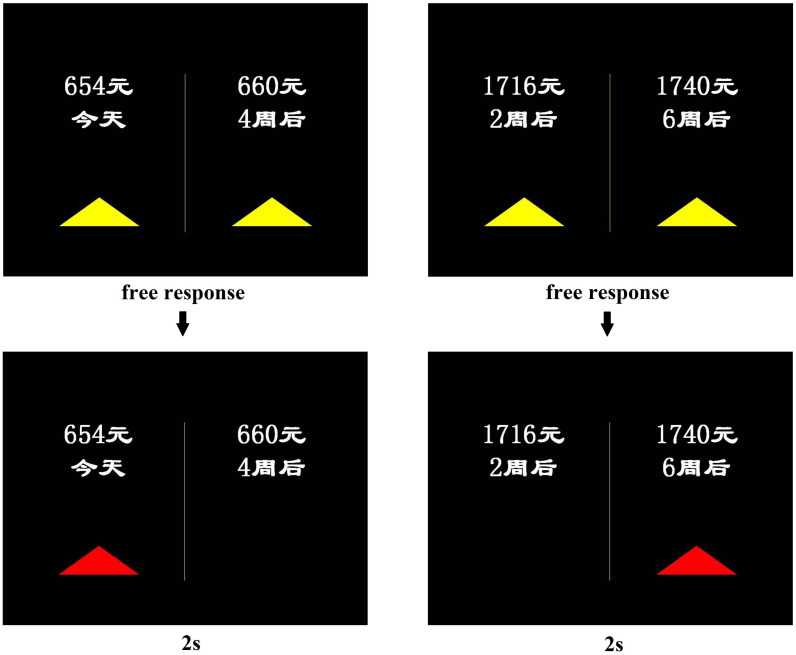
Examples of sooner reward and delayed reward options. During fMRI scanning, in each trial, the participants viewed a choice screen (above), made a self-paced choice response, and subsequently viewed a choice outcome screen (below) for 2 s. The reward options were in Simplified Chinese and read “
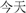
” (now), 2 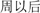 (2 weeks later), 4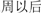 (4 weeks later), 6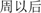 (6 weeks later).

The intertemporal binary choice paradigm (see Supplementary Material) was performed as described previously (McClure et al., 2004). In each pair of choices, the sooner option always had a lower reward value than the delayed option. The two options were separated by either 2 weeks or 1 month, with wait times ranging from the day of the experiment to 6 weeks later. Thus, the sooner option was sometimes available immediately and sometimes available after a delay (see Figure 2). The reward amount ranged from 31.25 to 250.00 RMB (USD equivalent, $5–40; conversion rate 1:6.25). In each experiment, the participant made 48 binary hypothetical choices, and the order of the choices was randomized within and across the participants.

The behavioral experiment was conducted entirely inside an fMRI scanner. The participants were instructed to make a series of choices between a smaller, sooner reward (*r1* available at delay *t1*) and a later, larger reward (*r2* available at delay *t2*; where *r1* < *r2* and *t1* < *t2*). Each participant was instructed to indicate her preference as soon as the choice was displayed by pressing one of two buttons that corresponded with the location of the preferred option with her right hand. The smaller, sooner options were always presented on the left. Decisions were self-paced with a maximum allowed reaction time of 15 s. After each choice, a feedback screen was presented for 2 s to indicate the choice outcome (McClure et al., 2004). All responses were submitted well before the 15-s time limit. Each subject completed two 24-trial runs. Each trial was followed by a jitter interval (1500 ms, 50% of trials; 4000 ms, 25%; 6000 ms, 16.7%; and 12,000 ms, 8.3%). Prior to being presented with the choices, the participants were administered two control questions to familiarize themselves with the nature of the task. The entire experiment took ∼ 8 min. The stimuli were presented via the *in vivo Esys* system for fMRI (Gainesville, FL, United States).

#### Imaging Data Acquisition

Scanning was conducted on a 3-T Siemens scanner at the fMRI laboratory of East China Normal University in Shanghai. Functional images were acquired with a gradient echo-planar imaging sequence, 2000-ms repetition time (TR), 30-ms echo time (TE), 220-mm field of view (FOV), 3 mm × 3 mm × 3.5 mm voxel size and 32 slices. 112 images per scan were acquired and a total was 224 across the two scans. The first five TRs acquired were discarded to allow for T1 equilibration. Prior to the fMRI scanning, a high-resolution structural image was acquired with a T1-weighted, multiplanar reconstruction sequence (TR = 1900 ms, TE = 3.42 ms, FOV = 256 mm, 1 mm × 1 mm × 1 mm voxel size, and 192 slices).

#### Behavioral Data Analysis

We employed a calculation method similar to that used by Kim et al. (2012), in which the behavioral data were fitted assuming a hyperbolic discount function. The discounted value function is given by

$$\begin{array}{c} V\left(r,t\right)=\frac{r}{1+kt} \end{array}$$

where *r* is the reward amount available at delay *t*, and *V* is the subjective value of the offer. The discount rate (*k*) for each subject was estimated by assuming a logistic decision function and maximizing the log-likelihood of the observed choices. Best-fit model parameters were determined in Matlab^1^ using a simplex search algorithm with 100 random initial parameter values. Subsequently, the *k* values were transformed logarithmically to permit parametric statistical analyses. For each participants, we calculated an immediate choice ratio (ICR; the number of sooner choices divided by the total number of choices). Because arcsine-square root transformed ICRs correlate well with the hyperbolic discounting rate variable *k* (Boettiger et al., 2009; Xu et al., 2009), we subjected the ICRs to arcsine-square root transformation.

Logarithmically transformed *k* values were compared between the late FP and mid-LP conditions with paired samples *t*-tests. We also conducted a correlation analysis between the transformed *k* and ICR values. The paired samples *t*-test was used to test for an order effect on the transformed *k* across the two menstrual phase conditions.

#### Imaging Data Analysis

The fMRI data were analyzed in Statistical Parametric Mapping 8 (SPM8, Wellcome Department of Cognitive Neurology, London, United Kingdom). We performed a slice-timing correction and, subsequently, aligned the data to correct for head movement. Images were smoothed with an 8-mm full-width at the half-maximum Gaussian kernel and then normalized to the Montreal Neurological Institute template and re-sampled at 3 mm × 3 mm × 3 mm resolution.

General linear modeling (GLM) was conducted in SPM8. For the first level GLM analysis, we used an event-related design to estimate neural responses to events of interest. Potentially confounding variables, such as trial-by-trial head movements and choice outcomes (i.e., motor responses), were included in the GLM as regressors of no interest. As suggested by the as soon as possible model (Kable and Glimcher, 2010), we expected participants to overweight the value of the soonest available reward regardless of whether it was offered immediately or after a delay. We pooled all smaller, sooner rewards (ignoring whether they were available immediately or at a delayed time) chosen by the participants in a pair together to form the sooner reward choice condition. The remaining larger, later rewards chosen by the participants were pooled together as the delayed choice condition. Each condition [sooner reward choice in the late FP (SFP), sooner reward choice in the mid-LP (SLP), delayed reward choice in the late FP (DFP), and delayed reward choice in the mid-LP (DLP)] were modeled as reaction times from the decision onset. In the first-level analysis, simple main effects were computed for each participant for each of the above mentioned conditions by applying a ‘1 0’ contrast, where 1 represents one of these conditions, and 0 represents all other possibilities.

For the second (group) level analysis, we conducted random effect modeling (flexible factorial design) to analyze the first-level individual contrast images. The main effect of menstrual phase was calculated by comparing late FP trials versus mid-LP trials. The main effect of delay discounting was obtained by comparing sooner-reward choice trials versus delayed-reward choice trials. The interaction between menstrual phase and delay discounting was calculated to extract brain regions that showed higher or lower sensitivity to the sooner reward outcome over the delayed reward outcome among women in the late FP versus women in the mid-LP.

All data were initially thresholded at a value of *p* < 0.001 (uncorrected), and the results were reported at a cluster statistical threshold of a family-wise error (FWE)-corrected *p* < 0.05. Activations were localized with the anatomy toolbox in SPM8 (Eickhoff et al., 2005) using the MRIcron automated anatomical labeling template (Tzourio-Mazoyer et al., 2002).

##### Region of interest (ROI) analyses

Region of interest analyses were conducted to further refine our hypothesis. ROIs were selected based on our hypotheses and previous studies (Cummings et al., 2014; Franklin et al., 2019). Based on prior relevant work (Pine et al., 2010), the following Montreal Neurological Institute (MNI) coordinates for bilateral ROIs were defined: putamen, MNI coordinates 30 −3 −12 and −24 12 −9; caudate, MNI coordinates 21 24 −3 and −9 12 9; and dlPFC, MNI coordinates 42 18 27 and −45 33 18. We extracted an average beta value for each ROI in each condition (sooner reward choice and delayed reward choice in each menstrual phase) for each participant by selecting a 6-mm sphere around the coordinates using the MarsBaR ROI toolbox 0.44 in SPM8 (Brett et al., 2002). All beta values were submitted to a 2 (delay discounting: sooner vs. delayed reward choice) × 2 (menstrual phase: FP vs. LP) × 6 (the aforementioned ROIs) mixed measures analysis of variance (ANOVA). We also completed a 2 (delay discounting: sooner vs. delayed reward choice) × 2 (menstrual phase: FP vs. LP) mixed measures ANOVA for each ROI. Mean beta values are reported with SDs.

##### Functional connectivity analysis

To test our neural circuitry hypothesis, we conducted beta series correlation (BSC) analyses of the functional connectivity between the DS (putamen and caudate) and dlPFC within each condition (SFP, SLP, DFP, and DLP). First, based on the above-defined ROIs, each trial was modeled as a separate event of interest (Rissman et al., 2004) and the beta series associated with each trial type within each ROI were extracted and sorted by study condition. Pair-wise BSC analyses were performed for the DS and the dlPFC. After calculating the correlation between activities in each ROI pair individually for each subject and for each condition across the time series, the correlation values obtained were subjected to Fisher transformation prior to being subjected to ANOVAs designed to detect which correlations varied across delay discounting choice and menstrual phase.

##### Correlations between DS-dlPFC functional connectivity and discount rate

We conducted correlation analyses between discount rate (transformed *k*) and functional connectivity (as indexed via BSCs) within each condition to investigate the effect of DS-dlPFC circuitry on impulsivity.

### Results

#### Demographics

A total of 8 participants were excluded from further analysis, including 3 for excessive head motion (>2-mm displacement in the *x*, *y*, or *z* dimension, or >2° angular shift) during scanning, 2 because tracking of their next menstruation revealed that we did not have an accurate menstrual date, and 3 whose behavioral data did not conform to the model from the log-likelihood of observed choices. The 16 remaining participants (mean age 22.44 ± 1.31 years; range, 20–24) included 8 participants who were initially scanned during their late FP, and 8 who were initially scanned during their mid-LP. The mean scores of the 16 participants in the final analyses had mean family economic and social status level scores of 5.81 ± 0.40 and 5.44 ± 0.51, respectively. There was very high agreement among the participants on subjective socioeconomic status [intra-class correlation (1,15) = 0.788], which suggests that behavioral effects observed in the experiment cannot be attributed to a confounding effect of subjective value variations.

#### Behavior

A paired samples *t*-test did not show an effect of testing order (i.e., which phase women were in during first test) on transformed *k* values [*t*_2_,_14_ = −0.97, *p* = 0.35, *d* = −0.52]. Meanwhile, a paired samples *t*-test showed a significant difference in transformed *k* values between the late FP and mid-LP (*t*_1_,_15_ = −2.14, *p* = 0.049 < 0.05, *d* = 0.43), with a significantly greater mean discount rate being observed in the late FP (−1.32 ± 0.49) than in the mid-LP (−1.51 ± 0.46) (Figure 1). Transformed *k* and ICR values were highly correlated within each menstrual phase (FP: *r* = 0.94, *p* < 0.001; LP: *r* = 0.96, *p* < 0.001) (see Table 1).

**TABLE 1:** Means and correlations of *k* and ICR values within menstrual phase datasets.

Statistical value	Late FP			Mid-LP		
	Mean (*n*)	SD	Pearson correlation *r*	Mean (*n*)	SD	Pearson correlation *r*
*k*	4.145 (16)	0.879		3.901 (16)	0.732	
ICR	0.544 (16)	0.208		0.478 (16)	0.180	
Transformed *k*	−1.323 (16)	0.488	0.938**	−1.506 (16)	0.458	0.964**
Transformed ICR	0.836 (16)	0.234		0.767 (16)	0.200	

** Correlation is significant at the 0.01 level (2-tailed).

#### Whole Brain Analysis

We observed main effects of menstrual phase on activity in several visual areas, including greater activation during the mid-LP than during the late FP in the bilateral lingual gyrus, bilateral calcarine gyrus, left middle occipital gyrus, and left inferior occipital gyrus. No other meaningful brain areas were found in the opposite contrast. With respect to delay discounting, brain regions that were preferentially activated by the prospect of a sooner reward choice over a delayed reward choice (sooner > delayed) included the right putamen, the right thalamus, and the right supplementary motor area. In the reverse contrast (delayed > sooner), greater activation was identified in the left postcentral gyrus and left superior parietal lobule. Analysis of interactions between menstrual phase and delay discounting revealed more active regions in the left putamen, bilateral caudate, bilateral visual areas [Brodmann area (BA)17 and 18], left hippocampus, and left insula in the (late FP – mid-LP) - (sooner – delayed) comparison; there was no regions that were significantly more active in the reverse comparison (Table 2).

**TABLE 2:** Brain regions whose activity is altered in relation to menstrual phase or delay discounting.

Corrected *p*	*k*	Regions of maxima peak	BA	*T*	H	MNI		
						*x*	*y*	*z*
**Interaction (late FP – mid-LP) - (sooner – delayed)**								
<0.001	317	Caudate nucleus		5.77	R	21	24	9
		Putamen	11	4.33	L	−15	15	−6
		Caudate nucleus	25	4.15	L	−6	12	5
<0.001	255	Calcarine gyrus	18	4.94	L	−3	−84	12
		Middle occipital gyrus	18	4.52	L	−18	−102	5
		Superior occipital gyrus	17	4.42	L	−9	−102	9
		Lingual gyrus	18	4.28	R	12	−84	−9
		Cuneus	18	4.02	R	12	−99	12
0.005	126	Hippocampus	20	4.86	L	−33	−9	−16
		Inferior temporal gyrus	20	4.82	L	−45	−9	−27
		Insula	48	3.44	L	−36	−9	−6
0.017	94	Cerebellum	37	4.79	L	30	−39	−23
0.018	92	Putamen	48	3.61	L	−21	6	−6
0.035	77	Postcentral gyrus	48	4.22	L	−60	−15	23
		Precentral gyrus	6	3.83	L	−60	3	30
**Main effect of menstrual cycle phase (mid-LP – late FP)**								
<0.001	911	Lingual gyrus	18	6.16	L	−12	−96	−13
		Inferior occipital gyrus	19	5.25	L	−36	−90	−13
		Calcarine gyrus	18	5.13	L	0	−99	9
		Middle occipital gyrus	17	5.09	L	−12	−99	5
		Inferior temporal gyrus	37	4.30	L	−45	−61	−8
0.032	79	Cerebellum		4.94	L	−6	−78	−37
0.034	61	Precentral gyrus	6	5.05	L	−51	−3	51
**Main effect of delay decision (sooner - delayed)**								
<0.001	1233	Precentral gyrus	4	10.68	R	36	−21	54
		Supplementary motor area	6	5.77	R	6	0	47
		Superior parietal lobule	5	5.24	R	15	−51	65
<0.001	704	Cerebellum	37	8.04	L	−21	−45	−27
		Lingual gyrus	18	3.93	L	−18	−72	−6
<0.001	304	Rolandic operculum	48	5.64	R	48	−18	19
		Thalamus		5.12	R	18	−21	9
		Putamen	48	4.11	R	30	−12	2
**Main effect of delay decision (delayed - sooner)**								
<0.001	709	Postcentral gyrus	4	10.72	L	−42	−27	65
		Superior parietal lobule	7	4.82	L	−24	−42	72
0.027	62	Cerebellum	19	6.08	R	15	−51	−16

*Results are reported at cluster significance after FWE correction at *p* < 0.05 for multiple comparisons; *k* = cluster size; L, left; R, right; H, hemisphere.*

#### ROI Analyses

**FIGURE 3:**
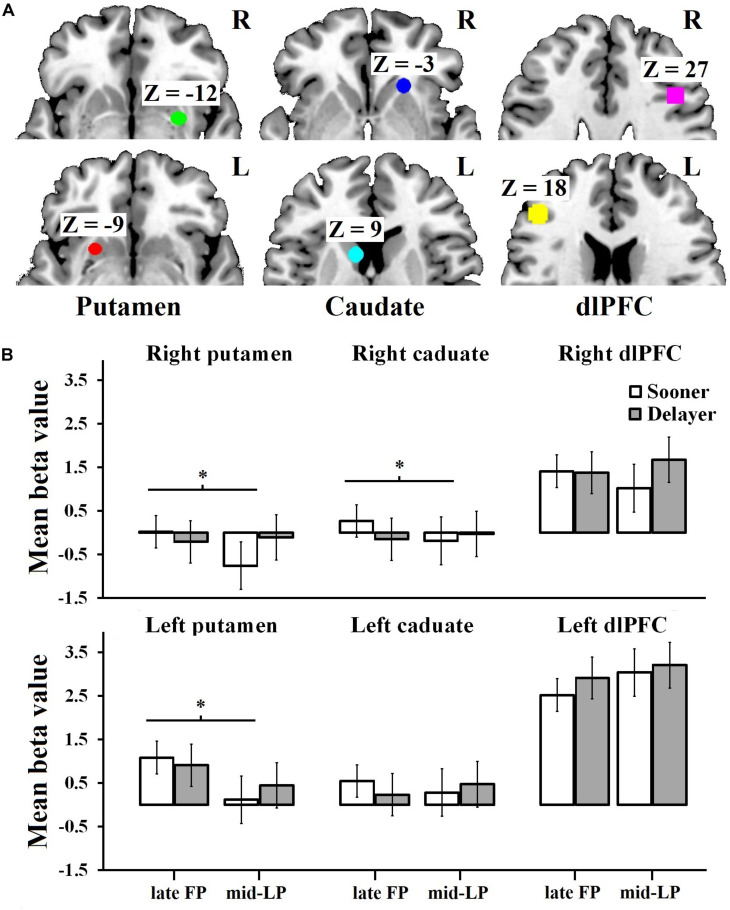
Mean beta values for ROIs by condition. **(A)** The ROIs of the left putamen [–24, 12, –9], right putamen [30, –3, –12], left caudate [–9, 12, 9], right caudate [21, 24, –3], left dlPFC [–45, 33, 18], and right dlPFC [42, 18, 27]. **(B)** Activation of the left putamen was significantly higher during the late FP than during the mid-LP. Moreover, when choosing sooner rewards, the mean beta values of the right putamen and the right caudate were much greater during the late FP than during the mid-LP. A significant interaction was observed for the right dlPFC; simple effects analysis showed a tendency for right dlPFC activation to be higher in the DLP condition than in the DFP, SLP, and SFP conditions. ^∗^The effect is significant at the 0.05 level.

A 2 (delay discounting) × 2 (menstrual phase) × 6 (ROI) mixed measures ANOVA revealed a significant main effect of ROI (*F*_5_,_90_ = 9.49, *p* < 0.001, $\eta_{\text{p}}^{2}$ = 0.35) and a significant interaction of delay discounting and menstrual phase (*F*_1_,_90_ = 8.97, *p* = 0.004, $\eta_{\text{p}}^{2}$ = 0.09) (Figure 3; non-significant effect data are reported in the Supplementary Material). Regarding the ROI effect, *post hoc* analysis indicated that the beta value for the left dlPFC was significantly greater than the values obtained for all other ROIs (*p*_*s*_ ≤ 0.005), while the beta value for the right dlPFC was significantly greater than the values obtained for the right putamen (*p* = 0.003) and right caudate (*p* = 0.010). Simple effects analysis of the delay discounting × menstrual phase interaction showed that when choosing the sooner rewards, beta values differed significantly between the late FP and mid-LP (*F*_1_,_90_ = 6.61, *p* = 0.010, $\eta_{\text{p}}^{2}$ = 0.07), with a greater mean beta value being observed in the late FP (0.98 ± 0.17) than in the mid-LP (0.58 ± 0.17). Specifically, for the left putamen, we found a significant main effect of menstrual phase (*F*_1_,_15_ = 5.1, *p* = 0.040, $\eta_{\text{p}}^{2}$ = 0.25), wherein women in the late FP (0.99 ± 1.22) had higher mean beta value than in the mid-LP (0.30 ± 0.77). There was a significant delay discounting × menstrual phase interaction for the right putamen (*F*_1_,_15_ = 5.02, *p* = 0.040, $\eta_{\text{p}}^{2}$ = 0.25). Simple effect analysis showed that the mean beta value was significantly higher when women chose sooner rewards in the late FP (0.02 ± 1.12) than when they did so in the mid-LP (−0.76 ± 0.72; *F*_1_,_15_ = 7.96, *p* = 0.010, $\eta_{\text{p}}^{2}$ = 0.35). No other effects were significant (Figure 3).

Similarly, we found a significant delay discounting × menstrual phase interaction for the right caudate (*F*_1_,_15_ = 6.86, *p* = 0.020, $\eta_{\text{p}}^{2}$ = 0.31). Simple effect analysis showed that the mean beta value was significantly higher when women chose the sooner rewards in the late FP (0.27 ± 0.77) than when they did so in the mid-LP (−0.19 ± 0.80; *F*_1_,_15_ = 8.65, *p* = 0.010, $\eta_{\text{p}}^{2}$ = 0.37). We did not find any significant effects for the left caudate (Figure 3).

For the right dlPFC, there was a significant delay discounting × menstrual phase interaction (*F*_1_,_15_ = 5.90, *p* = 0.030, $\eta_{\text{p}}^{2}$ = 0.28), but no significant effects were observed in the simple effects analysis. However, we did observe a tendency showing that the mean activation level in the DLP condition (1.67 ± 2.86) was higher than in the other conditions (SLP, 1.02 ± 2.28; SFP, 1.41 ± 1.91; DFP, 1.38 ± 2.34). No significant main effects (delay discounting, *F*_1_,_15_ = 1.32, *p* = 0.270, $\eta_{\text{p}}^{2}$ = 0.81; menstrual phase *F*_1_,_15_ = 0.53, *p* = 0.480, $\eta_{\text{p}}^{2}$ = 0.34) and no significant delay discounting × menstrual phase interaction (*F*_1_,_15_ = 0.33, *p* = 0.570, $\eta_{\text{p}}^{2}$ = 0.02) were observed for the left dlPFC (Figure 3).

The results above indicated that the right DS was more active during the late FP than during the mid-LP when sooner rewards were chosen (i.e., SFP > SLP for right DS). In contrast, the dlPFC showed a tendency to be more active during the mid-LP than during the late FP when delayed rewards were chosen (i.e., DLP > DFP for the dlPFC).

#### Functional Connectivity Across Menstrual Phases

An ANOVA revealed a significant main effect of menstrual phase on the functional connectivity between the left putamen and left dlPFC (*F*_1_,_15_ = 7.40, *p* = 0.020, $\eta_{\text{p}}^{2}$ = 0.33). The magnitude of this connectivity was significantly stronger in the mid-LP (0.22 ± 0.32) than in the late FP (0.00 ± 0.42). There were no other significant ROI functional connectivity effects.

#### Correlations Between DS-dlPFC Functional Connectivity and Discount Rate Across the Menstrual Phases

**FIGURE 4:**
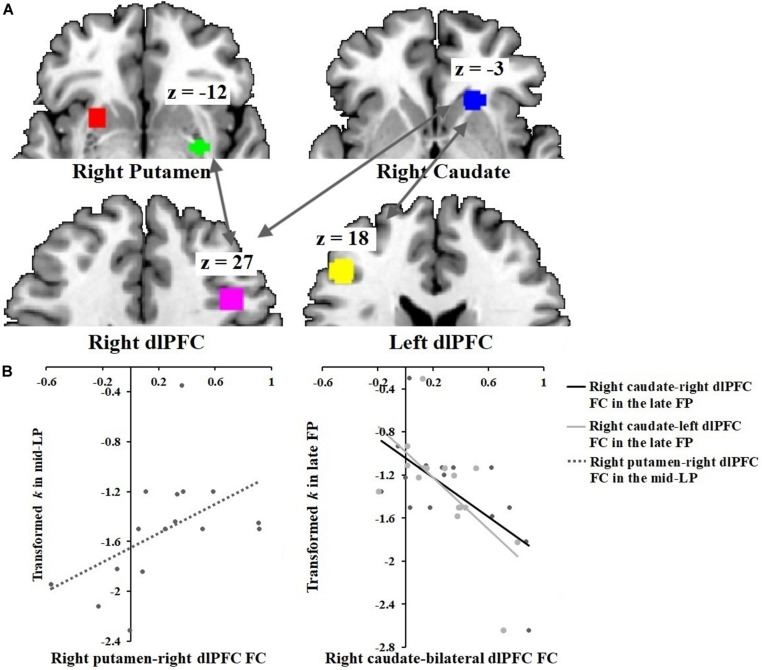
Correlations between DS-dlPFC functional connectivity and discount rate across menstrual phases. **(A)** ROIs: right putamen [30, –3, –12], right caudate [21, 24, –3], right dlPFC [42, 18, 27] and left dlPFC [–45, 33, 18]. **(B)** Discount rate correlated positively with right putamen-right dlPFC functional connectivity in the SLP condition. Discount rate correlated negatively with right caudate-bilateral dlPFC functional connectivity in the SFP condition.

In the SFP condition, functional connectivity between the right caudate and the bilateral dlPFC correlated inversely with discount rate (right caudate-right dlPFC: *r* = −0.64, *p* = 0.010; right caudate-left dlPFC: *r* = −0.64, *p* = 0.010) (Figure 4). Conversely, in the SLP condition, functional connectivity between the right dlPFC and the right putamen correlated directly with discount rate (*r* = 0.50, *p* = 0.050).

### Discussion

Using the delay discounting task, which assesses intertemporal choice similar to the task used by McClure et al. (2004), we demonstrated that delay discounting behavior in women was affected by menstrual phase, such that the discount rate was significantly greater in the late FP than in the mid-LP, and this augmented discount rate was associated with enhanced activity in the DS. Specifically, greater activation was observed in the left putamen during the late FP than during the mid-LP. Moreover, women showed greater activation in the right DS (putamen and caudate) when choosing sooner rewards in the late FP than when do so in the mid-LP (i.e., SFP > SLP), indicating that the right DS is more responsive to immediate rewards during the late FP than during the mid-LP. These results are consistent with the prior findings showing that a heightened drug cue-responsivity in women is associated with enhanced activity in the DS, especially in the putamen (Cummings et al., 2014; Franklin et al., 2019). Indeed, behavioral impulsivity has been linked to dopamine levels in the putamen. For example, in adult men, higher trait impulsivity as measured by Barratt Impulsiveness Scale (BIS) scores correlated negatively with dopamine transporter availability in the putamen (Costa et al., 2013). Additionally, a lower dopamine synthesis capacity in the putamen, indexed by 6-[^18^ F]fluoro-L-*m*-tyrosine signal, was shown to be predictive of an elevated Now bias and a reduced willingness to accept low-interest rate delayed rewards (Smith et al., 2016).

The VS/nucleus accumbens have been strongly implicated in the seeking the sooner rewards in intertemporal choice paradigms (McClure et al., 2004; Kim et al., 2012), with nucleus accumbens activation in particular being related to reward magnitude sensitivity (Ballard and Knutson, 2009). Our not finding an effect of menstrual phase on these areas suggests that VS reward sensitivity may not be modulated by menstrual phase. Indeed, intertemporal choice in humans has been shown to vary with region-specific dopamine processing, with regionally distinct associations with sensitivity to delay (the putamen) and reward magnitude (the VS) (Ballard and Knutson, 2009; Smith et al., 2016). Studies have linked dopamine availability in the putamen with time perception, a cognitive process thought to contribute to discounting of delayed rewards (Wittmann and Paulus, 2008; Takahashi, 2011; Smith et al., 2016). For example, Parkinson disease patients, who have deficits in putamen dopamine signaling, show selective impairments in time duration comparison (Dormal et al., 2012). A pharmaco-fMRI study related the relationship between dopamine depletion and time perception specifically to activity in the putamen (Coull et al., 2012). Our finding the putamen was more active in the SFP condition than in the SLP condition suggests that naturally cycled ovarian steroid hormones may modulate an aspect of time perception in intertemporal choice, perhaps through steroid hormone modulation of dopamine levels. However, proving such a modulatory effect would require dopamine level data. Future research should compare dopamine levels across menstrual phases to help elucidate the influence of steroid hormones on intertemporal choices.

Although we observed a significant menstrual phase × delay discounting interaction influence on right dlPFC activity, simple effect analysis revealed only a trend toward higher activation in the DLP condition relative the other conditions. *Post hoc* analysis of ROIs indicated that beta values were significantly higher for the dlPFC than for the DS. Because the striatum and prefrontal cortex are intermodulated via frontostriatal networks (Yuan et al., 2016), impulsivity variance across menstrual phases may be determined, at least in part, by relative activity levels between the DS and dlPFC. Although the activity in the dlPFC was stable across menstrual phases, heightened DS activity during sooner reward selection in women late FP may lead to relatively less potent top-down cognitive control. Conversely, lesser DS activity during the mid-LP may enable more potent cognitive control. Thus, our results suggest that the activities of the DS and dlPFC relative to each other may determine the level of cognitive control over impulsivity, such that there is stronger cognitive control during the mid-LP than during the late FP.

The present results also support the notion that DS-dlPFC circuitry is involved in the regulation of impulsivity. Specifically, when participants chose sooner rewards during the late FP, right caudate-dlPFC functional connectivity correlated negatively with discount rate, indicating a linear relationship between the reduced right caudate-dlPFC connectivity and increased impulsivity (indexed by discount rate) during the late FP, consistent with rs-functional connectivity findings in participants with internet gaming disorder (Yuan et al., 2016) and cigarette-dependent women (Wetherill et al., 2016). Conversely, in the SLP condition, right putamen-right dlPFC functional connectivity correlated directly with discount rate. The hierarchical reinforcement learning theory (Holroyd and Yeung, 2012) has suggested the dlPFC and motor structures in the DS (mainly the putamen) execute options chosen by other brain regions. If so, the aforementioned correlation may reflect an effect of execution function.

Anatomically, the DS includes the dorsal regions of the putamen and caudate nucleus (Porter et al., 2014). Although these two subregions both receive nigrostriatal dopaminergic projections (Fallon and Moore, 1978) and are involved in motivated behavior via the prefrontal cortex, functionally, the caudate is important for reward-related cognition whereas the putamen is more involved in motor behaviors (Baskerville and Douglas, 2010). Thus, the opposing directionality found in our results may reflect different functions of the DS-dlPFC circuitry.

Our results suggest that DS-dlPFC functional connectivity may modulate impulsivity in intertemporal choices, with opposing directionality and differential involvement of brain regions depending upon menstrual phase.

This study had notable limitations. First, although we hypothesized that, brain regions related to cognitive control would be more active during the mid LP, we did not find a significant main effect of menstrual phase on the activation of brain regions related to cognitive control. This negative finding could be related to inherent characteristics of cognitive involvement in the task (McClure et al., 2004), which could confound the effect of menstrual phase. Second, menstrual phase was not confirmed by any biological tests such as hormonal assays, potentially reducing phase designation accuracy. Previously studies have found that estimates based on the backward counting method, as used here, are correct 80–90% of the time (Carroll, 2018). Indeed, several participants were excluded after follow-up revealed inaccurate estimates. Third, the delay discounting task is hypothetical. Although previous studies have shown no significant differences behaviorally or neurologically between hypothetical and real reward outcomes (Johnson and Bickel, 2002; Madden et al., 2004; Lagorio and Madden, 2005; Bickel et al., 2009; Kim et al., 2012), it is possible that an extraordinarily impulsive person might much more easily forego a tantalizing immediate reward for a delayed larger reward in a hypothetical situation than in a real situation (Reynolds et al., 2006; Rosati et al., 2007; Jimura et al., 2009; Smits et al., 2013; Carroll, 2018). Fourth, the sample size of the present study was small, which weakens the strength of our results. Finally, that the smaller, sooner options were always presented on the left during scanning would influence our results on hemispheric asymmetry.

Use of the bilateral dlPFC as a seed can be a valuable tool for exploring the function of cognitive control networks in rs-fMRI studies (Cieslik et al., 2013; Hwang et al., 2015; Tao et al., 2017). Thus, to further probe whether there is increased cognitive control functioning during the mid-LP, relative to the late FP, and the involvement of DS-dlPFC circuitry in impulsivity regulation, we conducted a rs-fMRI experiment in the following accompanying study with a inclusion of hormonal assay confirmed menstrual phases.

## Hormone and rs-fMRI Study

### Methods

#### Participants

A cohort of 53 healthy, right-handed, female undergraduate participants (mean age ± SD, 22.77 ± 2.35 years; range 19–28) were recruited from a larger, common subject pool with certain inclusion criteria, namely: heterosexual sexual orientation; 28- to 30-day menstrual cycle; and no use of any form of hormones in the previous 3 months. The reason that the participants in the current study were exclusively heterosexual was the same as in the task fMRI study above. Therefore, the heterosexuality as an inclusion criteria was incidental (and otherwise irrelevant) for the research questions addressed in the current study. The study cohort included 28 women in their late FP (mean age, 22.54 ± 2.18 years) and 25 women in their mid-LP (mean age, 23.04 ± 2.54 years). Each woman was subjected to rs-fMRI. Late FP and mid-LP were considered to be 14–16 days and 6–8 days prior to the next predicted menstrual onset, respectively, determined as in Study 1. The testing order was randomized across participants and phases.

All participants had normal or corrected-to-normal vision. No participants reported a history of a psychiatric disorder or current use of psychoactive medications. The protocol was reviewed and approved by the Ethics Committee of the local university, and the study was conducted in accordance with the Declaration of Helsinki. Written informed consent was obtained from all participants, and they were compensated 50 RMB per hour.

#### Hormone Assays

A saliva sample was obtained from each participant immediately before scanning. To control for circadian influences on hormone levels, all experimental sessions were performed between 12:00 pm and 8:00 pm. Each participant drooled ∼2 mL of saliva passively into a collection tube, and each saliva sample was preserved in a refrigerator (−20°C). All samples were processed for estradiol and progesterone levels with DRG International ELISA kits and the ELISA results were measured with a Thermo Devices Multiskan MK3 by ThermoFisher Scientific Shanghai Company. One-way ANOVAs were conducted on each hormone separately to verify cycle phases with sample collection time as the covariate.

#### BIS-11

After finishing the saliva sample collection, participants completed the BIS-11 (Patton et al., 1995). The BIS-11 is a self-report questionnaire containing 30 items divided into three subscales: (i) attentional impulsiveness (e.g., “I am a careful thinker”); (ii) motor impulsiveness (e.g., “I do things without thinking”); and (iii) non-planning impulsiveness (e.g., “I am more interested in the present than the future”). Each item was scored from 1 (*strongly disagree*) to 4 (*strongly agree*), with higher scores indicating higher levels of impulsivity.

#### Image Acquisition

Previously, rs-functional connectivity has been used to analyze neural circuitry dynamics in normal populations (Choi et al., 2012) and in pathological states (Fox and Greicius, 2010). The rs-fMRI scanning was conducted in a 3-T Siemens scanner at our institution’s fMRI facility. Functional images were acquired with a gradient echo-planar imaging sequence, 2000-ms TR, 30-ms TE, 384-mm FOV, 3 mm × 3 mm × 3.5 mm voxel size, and 33 slices. The images associated with the first ten repetitions were discarded to allow for T1 equilibration. Participants were instructed to relax with their eyes open during scanning, which lasted about 8 min. Prior to fMRI scanning, a high-resolution structural image was acquired with a T1-weighted, multiplanar reconstruction sequence (TR = 2530 ms, TE = 2.98 ms, FOV = 256 mm, 1 mm × 1 mm × 1 mm voxel size, and 192 slices).

#### Preprocessing

Preprocessing was performed in advanced DPARSF module V3.2 software. After correcting all volume slices for varying signal acquisition times, the images in each participant’s series were realigned. Individual structural images were then co-registered to the mean functional image. The transformed structural images were segmented into gray matter, white matter, and cerebrospinal fluid (Ashburner et al., 2005). Friston’s 24-parameter model was utilized to regress out head motion signal artifacts from the realigned data. White matter and cerebrospinal fluid signals were regressed out to reduce respiratory and cardiac effects. Spatial smoothing (4-mm full-width at half-maximum kernel) were applied to the functional images.

#### Independent Component Analysis (ICA)

GIFT (Group ICA of fMRI Toolbox) (Calhoun et al., 2001) was used to conduct group-level ICA. In the pre-processing step, datasets from each individual were mean corrected by subtracting the image mean per time point. Thereafter, for each participant, dataset dimensionality was reduced with principal component analysis using the default setting. Then, the data were group concatenated and subjected to two further principal component analysis data reduction steps. Next, the infomax algorithm was used to estimate 53 independent components from the reduced data. Finally, individual spatial maps were back-constructed from group-level component estimates with group ICA. The time courses and values of each map were scaled to represent percent signal change. No temporal filtering was applied to the data in GIFT.

Networks of interest were identified from the 53 components by spatial sorting. The executive control network (ECN) was identified by spatial sorting and statistical comparison to the resting state network 2 (RSN2) described by Mantini et al. (2007), which is strongly associated with goal-directed stimulus-response selection. RSN2 is comprised of areas within the bilateral intraparietal sulcus and at the intersections of the precentral and superior frontal sulcus, ventral precentral cortex, and middle frontal gyrus regions (Mantini et al., 2007). An RSN2 mask was constructed in WFU_PickAtlas 3.0 (Berry et al., 2015). Correlations between ECN components and RSN2 components were calculated and reported with the standard of *r* ≥ 0.30 (Weis et al., 2017).

An independent sample *t*-test was carried out on the group level to estimate the effect of menstrual phase (late FP vs. mid-LP) on the beta weights of each identified component of the ECN in SPM8. Initially, the data were thresholded at *p* < 0.001 (uncorrected); the results are reported at a cluster statistical threshold of *p* < 0.05 (FWE-corrected). Activations were localized based on the MRIcron automated anatomical labeling template (Tzourio-Mazoyer et al., 2002).

#### Correlations Between Hormone Levels and Activity in Brain Regions

After preprocessing, the amplitude of low-frequency fluctuations of the BOLD signal (ALFF), which is thought to be related to regional spontaneous neural activity, was used to identify differences in regional resting cerebral function between menstrual phases (Cordes et al., 2001). After bandpass filtering (0.01–0.08 Hz), white matter and cerebrospinal fluid signals were removed. Following linear detrending, voxel-wise time series were transformed to the frequency domain by fast Fourier transformation to obtain power spectra. The ALFF measure at each voxel represents the square root of the power across a low-frequency range. The ALFF of each voxel was z-transformed for each subject to standardize the data to allow inter-subject comparisons. Multiple regressions of estradiol, progesterone, and relative estradiol [calculated as (estradiol - progesterone)/progesterone], and relative progesterone [calculated as (progesterone - estradiol)/estradiol] levels on z-transformed ALFF data were conducted for each menstrual phase to verify hormone-correlated brain activity.

#### Correlations Between Behavioral Impulsivity and DS-dlPFC Functional Connectivity

The relationship between the impulsivity and DS-dlPFC functional connectivity was investigated by correlation analyses. We defined ROIs (bilateral putamen/caudate and dlPFC) and analyzed the functional connectivity between these ROIs from each participant’s preprocessed data using the coordinates and methods described for the task fMRI study above. Regression analyses were conducted on DS-dlPFC functional connectivity data relative to each BIS-11 subscale score for each menstrual phase.

### Results

#### Demographics

Two participants were excluded for excessive movement during scanning (≥2-mm maximum displacement in the *x*, *y*, or *z* dimension; or ≥2° angular motion). Two participants were excluded due to incorrect menstrual date estimation discovered upon tracking the next menstrual cycle. Of the 49 remaining participants (mean age, 22.86 ± 2.29 years; range, 19–28), 25 were scanned during their late FP (mean age, 22.52 years ± 2.38), and 24 were scanned during their mid-LP (mean age, 23.21 ± 2.19). Age did not differ significantly between the two menstrual phase groups (*t*_2_,_47_ = 1.05, *p* = 0.30, *d* = 0.31).

#### Hormone Assays

Hormone concentrations for each menstrual phase group are reported in Table 3. A one-way ANOVA with collection time as a covariate confirmed significantly higher progesterone levels in the mid LP group than in the late FP group (*F*_1_,_46_ = 4.16, *p* = 0.047, $\eta_{\text{p}}^{2}$ = 0.08). Meanwhile, estradiol levels were similar between these two groups (*t*_2_,_49_ = 0.17, *p* = 0.68, $\eta_{\text{p}}^{2}$ = 0.004).

**TABLE 3:** Mean (± SD) estradiol and progesterone levels.

Hormone	Menstrual phase	
	Late FP	Mid-LP
Estradiol, pg/ml	4.52 ± 2.50	6.27 ± 3.66
Progesterone, pg/ml	28.10 ± 33.39	170.33 ± 130.38

#### ECN

**FIGURE 5:**
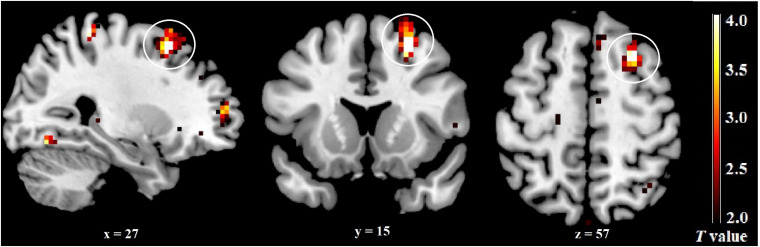
Differences in the ECN across menstrual phases. The right dlPFC was significantly more activated during the mid-LP than during the late FP.

An independent sample *t*-test revealed a significant difference between the late FP and the mid-LP groups for component 50 activity. Higher activity was observed in the right dlPFC (superior frontal gyrus, BA 8, *p* = 0.050, FWE-corrected, *k* = 20) during the mid-LP than during the late FP (Table 4 and Figure 5). No other significant differenced were found between the menstrual phases for the activities of any other ECN components.

**TABLE 4:** Brain regions whose activity is altered in relation to menstrual phase in the ECN.

Corrected *p*	*k*	Regions of maxima peak	BA	*T*	H	MNI		
						*x*	*y*	*z*
**Mid-LP – late FP**								
0.05	20	Superior Frontal Gyrus	8	5.28	R	27	15	57

*Results are reported at Cluster significant after FWE correction at *p* < 0.05 for multiple comparisons; *k* = cluster size, MNI, Montreal Neurological Institute. L, left; R, right; H, hemisphere; BA, Broca’s area.*

#### Correlations Between Hormone Levels and Brain Region Activity in Whole-Brain Analysis

During the late FP, no significant correlations were found between absolute levels of estradiol or progesterone and the observed ALFF in brain regions. There was a significant positive correlation between the relative progesterone level and the ALFF in areas within the right hippocampus, thalamus, precuneus, and left angular gyrus. During the mid-LP, there was a significant positive correlation between absolute estradiol level and ALFF of brain areas within the bilateral dlPFC and superior medial prefrontal cortex. Relative estradiol level correlated with the ALFF of brain regions in the right dlPFC and left postcentral gyrus as well. Relative progesterone level correlated with the ALFF of brain areas within the right superior temporal and middle temporal cortices (Table 5).

**TABLE 5:** Correlations between hormone levels and brain activity.

Corrected *p*	*k*	Regions with maxima peaks	BA	*T*	H	MNI		
						*x*	*y*	*z*
**Positive correlations of relative PROG with brain regions in the late FP**								
0.015	29	Hippocampus	29	8.85	R	12	−39	9
		Thalamus		6.54	R	15	−30	9
		Precuneus	27	4.23	R	21	−39	3
0.045	23	Angular	39	6.63	L	−45	−54	27
**Positive correlations of E2 with brain regions in the mid-LP**								
<0.001	109	Superior frontal gyrus	11	7.58	R	21	72	0
		Middle frontal gyrus	46	5.66	R	45	57	12
		Medial superior frontal gyrus	10	5.66	R	15	72	9
<0.001	54	Middle frontal gyrus	46	6.12	L	−48	51	−3
**Positive correlations of relative E2 with brain regions in the mid-LP**								
0.019	27	Postcentral gyrus	43	6.43	L	−60	−15	36
0.034	24	Superior frontal gyrus	6	5.61	R	24	−9	66
**Positive correlations of relative PROG with brain regions in the mid-LP**								
<0.001	22	Superior temporal gyrus	22	5.17	R	72	−30	9
		Middle temporal gyrus	22	5.00	R	72	−39	3

*Results are reported at Cluster significant after FWE correction at *p* < 0.05 for multiple comparisons; *k* = cluster size; L, left; R, right; H, hemisphere.*

#### ROI Based rs-Functional Connectivity Correlations With BIS-11

**FIGURE 6:**
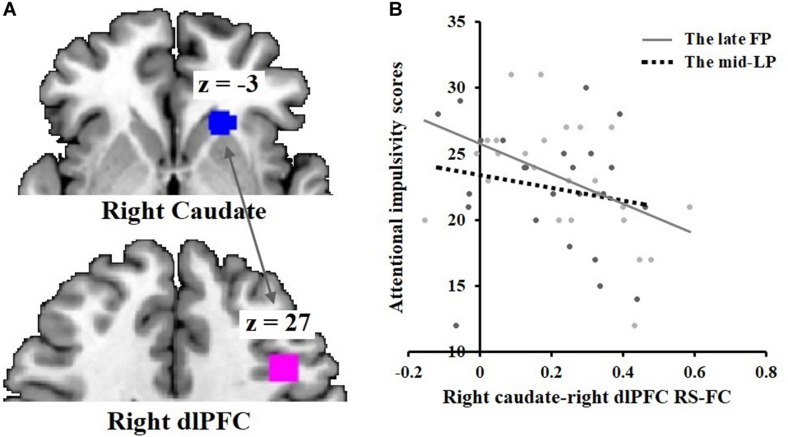
Negative correlation between attentional impulsivity and DS-dlPFC functional connectivity in the late FP. **(A)** Rs-functional connectivity between the right caudate [21, 24, –3] and right dlPFC [42, 18, 27]. **(B)** Negative correlation of attentional impulsivity with right caudate-right dlPFC rs-functional connectivity in the late FP; no such effect was observed in the mid-LP.

A significant negative correlation was found between BIS-11 attentional impulsivity subscale scores and rs-functional connectivity between the right caudate and right dlPFC (*r* = −0.47, *p* = 0.020) during the late FP (Figure 6). No significant correlations were found during the mid-LP.

### Discussion

Hormone assays confirmed the menstrual phases (late FP, mid-LP) predicted by the backward counting method. In the rs-fMRI study, the right dlPFC (superior frontal gyrus) was significantly more active during the mid-LP than during the late FP, consistent with our hypothesis of there being greater cognitive control functioning in the mid-LP than in the late FP.

The magnitude of right caudate-right dlPFC rs-functional connectivity correlated negatively with BIS-11 attentional impulsivity subscale scores during the late FP, consistent with the above reported findings in our delay discounting task-based experiment in study 1. No positive correlations between rs-functional connectivity and impulsivity were observed.

Our finding of positive correlations of dlPFC activity with absolute or relative estradiol levels during the mid-LP suggests that augmented cognitive control function during the mid-LP may be accounted for by the relative levels of estradiol to progesterone, wherein both hormones are elevated. Previous work has shown that a change in the Now bias in intertemporal choices correlates inversely with changes in estradiol levels, decreasing from the low estradiol period of the menstrual phase (estradiol nadir is during menstruation) to the late FP (estradiol levels increase gradually in the early FP then increase rapidly until peaking in the late FP) (Smith et al., 2014). The present results further suggest that it may be the relative level of estradiol that modulates cognitive control of impulsivity during the mid-LP. Consistent with this possibility, the induction of several adaptive behaviors associated with gonadal hormones in intact animals have been shown to require concurrent changes in circulating levels of estradiol and progesterone (Tennent et al., 1980). It is possible that the lack of significant correlations between estradiol levels and activities of brain regions during the late FP in this study were due to there being a quite narrow range in hormone level variabilities (Smith et al., 2014).

On the other hand, our correlation analysis indicated that brain regions related to social cognition, especially in relation to theory of mind (Gallagher and Frith, 2003), are particularly sensitive to the relative levels of progesterone during the mid-LP. Higher progesterone levels in humans have been reported to be associated with greater motivation for affiliation (Schultheiss et al., 2004); whereas, cooperative tendencies have been shown to correlate negatively with estimated estradiol levels (Anderl et al., 2015). From an evolutionary perspective, it is noteworthy that the mid-LP is the phase when women’s progesterone levels are highest and women’s reproductive systems are preparing for possible pregnancy (Maner and Miller, 2014). Thus, the activation of brain regions related to social cognition and theory of mind may provide advantages for pregnant women.

## General Discussion

In the presently reported task-based fMRI and rs-fMRI studies, we demonstrated that circulating gonadal steroid hormones in women affected cognitive control of impulsivity, such that women had greater cognitive control on impulsivity during the mid-LP than during the late FP. Neurophysiologically, the DS and dlPFC are the main brain regions involved in cognitive control of impulsivity via the DS-dlPFC neural circuit. Specifically, right dlPFC activity was significantly stronger relative to the activity of DS during the mid-LP than during the late FP, and right dlPFC activity was sensitive to relative estradiol levels during the mid-LP. This cognitive advantage during the mid-LP may have evolutionary roots. As mentioned above, from an evolutionary perspective, the immediate goal of women in their mid-LP would be protection of their potential baby. The present finding of relative progesterone levels correlating positively with brain regions involved in social cognition provides direct evidence for this proposed goal. Thus, according to hierarchical reinforcement learning theory (Holroyd and Yeung, 2012), women keeping a higher level of cognitive control function would be adaptive for facilitating the realization of these goals by fulfilling a variety of women’s specific social goals during their mid-LP.

On the other hand, the DS was significantly more responsive to reward stimuli in the late FP, the phase associated with weak cognitive control, than it was during the mid LP. This change in neural responsiveness could also serve the evolutionary goal of mating. Evidence has shown that menstrual phase influences women’s cognitive control in relation to male faces but not in relation to female faces in a manner that appears to reflect their potential fertility during the late FP (Roberts et al., 2009). Indeed, a number of studies have shown increased impulsive behavior in relation to mating motivation in the late FP relative to the mid-LP. For example, women have been shown to exhibit enhanced impulsivity favoring the selection of sexier clothing and accessories near ovulation (Durante et al., 2008, 2011; Zhuang and Wang, 2014), especially under the priming condition of mating motivation (Zhuang and Wang, 2014). Also, women have been found to be more mobile and socially active in contexts related to mating motivation (Fessler and Navarrete, 2003; Miller et al., 2007).

## Limitations, Implications, and Future Researches

The current findings indicate that ovarian hormones impact impulsive behaviors and that relative estradiol and progesterone levels may modulate the relationship between cognitive control and impulsivity. These results and their underlying neural basis have important implications for understanding the neural mechanisms that mediate impulsive control in a variety of contexts, including drug abuse (Cummings et al., 2014), postpartum psychosis, which is associated with drastic hormonal changes and a loss of inhibition (Ahokas et al., 2000), and borderline personality disorder, which has been associated with a variance in aggressive and impulsive behaviors over different menstrual phases (Dougherty et al., 1999). Our findings may provide novel directions for the treatment of these disorders and others, including attention deficit hyperactivity disorder, Parkinson disease, and obsessive-compulsive disorder. For example, manipulating the hormonal milieu may be helpful for alleviating these disorders and diseases. Treatment during the mid-LP may be particularly effective and important for attenuating hyperimpulsivity.

The present findings should be considered in the context of two notable limitations. Firstly, the sample size in the task-based fMRI study was relatively small, which may have weakened the power of and level of significance in our results. Secondly, the hormone and rs-fMRI study did not include direct assessments of dopamine levels across the menstrual phases, which limits our ability to make conclusions regarding a direct relationship between dopaminergic pathway activity and the observed difference in impulsive behavior between menstrual phases. Determination of dopamine levels at different points within the menstrual cycle in future research would help to provide a more detailed understanding of the influence of female gonadal hormones on impulsive behaviors.

In conclusion, natural menstrual cycle phase affected cognitive control of impulsivity. Women were more apt to postpone receiving rewards during the mid-LP than during the late FP. Our fMRI findings support the possibility that menstrual phase-associated behavioral changes may be consequent to hormonally induced alterations in the dlPFC and DS and the communication between these brain regions.

## Data Availability Statement

The raw data supporting the conclusions of this article will be made available by the authors, without undue reservation.

## Ethics Statement

The studies involving human participants were reviewed and approved by Committee on Human Research Protection, East China Normal University. The patients/participants provided their written informed consent to participate in this study.

## Author Contributions

J-YZ conceived and designed the experiments. J-XW, QL, and MF performed the experiments. J-XW analyzed the data. J-YZ wrote the manuscript. All authors contributed to the article and approved the submitted version.

## Conflict of Interest

The authors declare that the research was conducted in the absence of any commercial or financial relationships that could be construed as a potential conflict of interest.

## Footnotes

1 https://www.mathworks.com/
