# Supplementary material for: Neural Basis of Increased Cognitive Control of Impulsivity During the Mid-Luteal Phase Relative to the Late Follicular Phase of the Menstrual Cycle
Source: Front Hum Neurosci. 2020 Nov 12;14:568399. doi: 10.3389/fnhum.2020.568399 (PMC7693576; doi:10.3389/fnhum.2020.568399)
Supplement: Supplementary file 1 [file Table_1.DOCX]

| Effect | *F* | *df*_1_ | *df*_2_ | Sig. | **η***_p_^2^* |
| --- | --- | --- | --- | --- | --- |
| Menstrual phase | 1.185 | 1 | 90 | 0.279 | 0.013 |
| Delay discounting | 1.440 | 1 | 90 | 0.233 | 0.016 |
| ROI | 9.489 | 5 | 90 | 0.000 | 0.345 |
| Menstrual phase ×ROI | 1.292 | 5 | 90 | 0.274 | 0.067 |
| Delay discounting×ROI | 0.613 | 5 | 90 | 0.690 | 0.033 |
| Menstrual phase×Delay discounting | 8.971 | 1 | 90 | 0.004 | 0.091 |
| Menstrual phase×Delay discounting×ROI | 0.894 | 5 | 90 | 0.489 | 0.047 |

Results of the 2 delay discounting × 2 menstrual phase × 6 ROIs measures of analysis.
