# Supplementary material for: Neural Basis of Increased Cognitive Control of Impulsivity During the Mid-Luteal Phase Relative to the Late Follicular Phase of the Menstrual Cycle
Source: Front Hum Neurosci. 2020 Nov 12;14:568399. doi: 10.3389/fnhum.2020.568399 (PMC7693576; doi:10.3389/fnhum.2020.568399)
Supplement: Supplementary file 2 [file Table_2.DOCX]

Table of choices. The actual testing order was random. The unit of the choice was “yuan ” in Chinese money.

| Trial | Choice |  |  |  |
| --- | --- | --- | --- | --- |
|  | Today | 2 weeks later | 4 weeks later | 6 weeks later |
| 1 | 624 | 660 |  |  |
| 2 | 1248 | 1860 |  |  |
| 3 | 1572 |  | 1620 |  |
| 4 | 1104 |  | 1680 |  |
| 5 |  | 1488 | 1500 |  |
| 6 |  | 1038 | 1560 |  |
| 7 |  | 1716 | 1740 |  |
| 8 |  | 936 | 1380 |  |
| 9 | 1050 | 1080 |  |  |
| 10 | 990 | 1020 |  |  |
| 11 | 912 | 960 |  |  |
| 12 | 1500 | 1680 |  |  |
| 13 | 1410 | 1620 |  |  |
| 14 | 2040 | 2520 |  |  |
| 15 | 798 | 1080 |  |  |
| 16 | 1284 | 1920 |  |  |
| 17 | 654 |  | 660 |  |
| 18 | 1668 |  | 1740 |  |
| 19 | 738 |  | 780 |  |
| 20 | 1278 |  | 1380 |  |
| 21 | 1470 |  | 1680 |  |
| 22 | 480 |  | 600 |  |
| 23 | 1530 |  | 2040 |  |
| 24 | 1344 |  | 2040 |  |
| 25 |  | 1158 | 1200 |  |
| 26 |  | 906 | 960 |  |
| 27 |  | 1314 | 1380 |  |
| 28 |  | 1332 | 1440 |  |
| 29 |  | 774 | 900 |  |
| 30 |  | 1542 | 1920 |  |
| 31 |  | 1710 | 2280 |  |
| 32 |  | 804 | 1200 |  |
| 33 |  | 1224 |  | 1260 |
| 34 |  | 1884 |  | 1920 |
| 35 |  | 1026 |  | 1080 |
| 36 |  | 972 |  | 1080 |
| 37 |  | 1152 |  | 1320 |
| 38 |  | 1326 |  | 1680 |
| 39 |  | 834 |  | 1140 |
| 40 |  | 936 |  | 1380 |
| 41 |  |  | 1938 | 1980 |
| 42 |  |  | 1212 | 1260 |
| 43 |  |  | 564 | 600 |
| 44 |  |  | 726 | 780 |
| 45 |  |  | 600 | 720 |
| 46 |  |  | 1842 | 2280 |
| 47 |  |  | 1530 | 2040 |
| 48 |  |  | 1926 | 2880 |
